# Planned parathyroidectomy: the new standard in hypercalcemic crisis

**DOI:** 10.20945/2359-3997000000613

**Published:** 2023-05-10

**Authors:** Murilo Catafesta das Neves, Marcello Rosano, Monique Nakayama Ohe, Giulianno Molina de Mello, Davi Knoll Ribeiro, Rodrigo Oliveira Santos

**Affiliations:** 1 Universidade Federal de São Paulo Departamento de Otorrinolaringologia e Cirurgia de Cabeça e Pescoço São Paulo SP Brasil Departamento de Otorrinolaringologia e Cirurgia de Cabeça e Pescoço, Universidade Federal de São Paulo, São Paulo, SP, Brasil; 2 Universidade Federal de São Paulo Departamento de Endocrinologia São Paulo SP Brasil Departamento de Endocrinologia, Universidade Federal de São Paulo, São Paulo, SP, Brasil

**Keywords:** Hypercalcemia, primary hyperparathyroidism, parathyroidectomy

## Abstract

**Objective:**

The study sought to determine the clinical features of hyperparathyroid-induced hypercalcemic crisis (HIHC) along with treatment options and outcomes.

**Subjects and methods:**

This is a retrospective analysis of our historical cohort of patients with primary hyperparathyroidism (PHPT). Patients were divided in groups according to their calcium levels and clinical presentation. HIHC (group 1) was assumed when patients had high calcium levels and needed emergency hospitalization. Group 2 was composed of patients with calcium levels above 16 mg/dL or patients who needed hospitalization for classical PHPT symptoms. Group 3 was composed of clinically stable patients with calcium levels between 14 and 16 mg/dL, who were electively treated.

**Results:**

Twenty-nine patients had calcium levels above 14 mg/dL. HIHC group had seven patients, and initial clinical measures had good response in two patients, moderate response in one patient, and poor response in four patients. All poor responders underwent immediate surgery, and one of them died due to HIHC complications. Group 2 had nine patients, and all were successfully treated during hospitalization. Group 3 had 13 patients, and all had a successful elective surgery.

**Conclusion:**

HIHC is a life-threatening condition that requires fast clinical intervention. Surgery is the only definitive treatment and should be planned for all patients. Poor response to initial clinical measures should direct treatment toward surgery to avoid disease progression and clinical deterioration.

## INTRODUCTION

Primary hyperparathyroidism (PHPT) is a hypercalcemic metabolic disorder related to high or inappropriately normal levels of PTH ( 1 ). Classical clinical symptoms may include renal, skeletal, and gastrointestinal manifestations, whereas non-classical features such as cardiovascular, neuropsychiatric, cognitive, and musculoskeletal symptoms can still be prevalent ( 1 , 2 ).

Over the years, the progressive awareness about PHPT physiology has promoted a shift toward a more oligosymptomatic or completely asymptomatic clinical presentation, when only biochemical abnormalities are present at the time of diagnosis ( 2 ).

So, at present, the less frequent clinical presentation is those of highly symptomatic patients, particularly the ones related to hypercalcemic crisis ( 3 ). Despite the lacking standard definition, hyperparathyroid-induced hypercalcemic crisis (HIHC) is considered when albumin-correct serum calcium level is greater than 14 mg/dL along with multi-organ dysfunction in patients with PHPT ( 4 , 5 ).

The fast deterioration of central nervous system and cardiac, gastrointestinal, and renal functions leads to an emergency hospitalization ( 3 – 6 ). This life-threatening condition requires immediate recognition and aggressive measures to reduce serum calcium ( 6 ). But despite this initial therapy, refractory cases will demand an immediate parathyroidectomy ( 3 – 6 ).

In our country, where most depend on the public health system, there are large difficulties in accessing specialized centers. Patients take years to be properly treated. Therefore, we see very advanced cases of PHPT. This study sought to determine the features of HIHC and its incidence, clinical presentation, and treatment options, along with long-term outcomes, in our surgically treated cohort.

## SUBJECTS AND METHODS

This is a retrospective analysis of our historical cohort of patients treated between 2000 and 2021. All medical records and patients’ charts were reviewed. Our institution is a specialized tertiary public health hospital that receives many patients referred only for surgical treatment of PHPT. The time of diagnosis was determined by the first observation of PTH-related hypercalcemia on laboratory exams.

For the purpose of considering HIHC, a calcium level of 14 mg/dL was adopted as a threshold. All patients with calcium levels above 14 mg/dL had all demographic and laboratorial data, along with clinical and surgical treatment information collected for analysis. Patients with calcium levels below 14 mg/dL were excluded from further analysis, being considered only for the determination of HIHC incidence.

To understand the dynamics of HIHC in our population, patients were divided among different groups as follows:

- Group 1 patients are those who were admitted at the emergency and hospitalized due to acute calcium intoxication symptoms, such as polyuria, polydipsia, nausea, vomiting, muscle weakness, confusion, lethargy, arrhythmia, hypotension, and shock. This is the HIHC group.
- Group 2 patients are those who had no symptoms related to HIHC (no multi-organ dysfunction) but still needed hospitalization due to severe PHPT classical symptoms or had calcium levels > 16 mg/dL to prevent further clinical deterioration. This is the group of severe PHPT.
- Group 3 patients are those who had no symptoms related to HIHC or severe PHPT (could not be included in the previous groups) and had calcium levels between 14 and 15.9 mg/dL.

For surgical treatment, localization exams were performed according to the institutional protocol and patients’ clinical conditions. Whenever possible, ultrasound and sestamibi were both performed. Severely ill and hospitalized patients undergone ultrasound and/or computed tomography.

The response to initial clinical measurements was adopted as follows: good response – when patient had full clinical recovery and calcium levels dropped below 14 mg/dL; moderate response – when patient had a moderate clinical recovery or calcium levels were consistently above 14 mg/dL; and poor response – when patient had no clinical improvement or calcium levels were above 16 mg/dL.

RStudio was used to analyze variables, and analysis of variance (ANOVA) was used to determine differences between groups. Data are expressed as absolute or mean ± standard deviation along with minimum and maximum values; *P* < 0.05 was considered significant. Review board approval number over the cohort follow-up: 886/2000, 0234/2006, 0354/2009, and 0194/2021.

## RESULTS

Our cohort had 399 patients; 370 of them had calcium levels below the threshold, and 29 (7.3%) patients had calcium levels above 14 mg/dL. The distribution of these 29 patients among groups, their demographic information, the presence of classical symptoms, and laboratorial and surgical information are presented in Table 1 .

**Table 1:** Demographic information, symptoms, and laboratorial and surgical information of all 29 patients with calcium levels above 14 mg/dL

			Group 1	Group 2	Group 3
N		29	7	9	13
Female		15	5	5	5
Age (years)		48.4 (11-73)	57 (29-44)	42.5 (28-69)	47.9 (11-66)
Symptoms					
	Renal stones	18	2	7	9
	Low BMD	10	2	1	7
	Fracture/brown tumor	6	0	6	0
Diagnosis at hospitalization		11	7	4	0
Total calcium		15.6 ± 1.7 (14.0-20.2)	17.4 ± 2.2 (14.3-20.2)	15.7 ± 1.4 (14.6-19.3)	14.5 ± 0.25 (14.0-14.9)
Ionized calcium		2.14 ± 1.8 (1.8-2.97)	2.41 ± 0.40 (1.87-2.97)	2.18 ± 0.21 (1.9-2.5)	1.97 ± 0.12 (1.80-2.19)
PTH		1,226.6 ± 839.4 (118-3,402)	1,772.3 ± 1,029.4 (248-3,402)	1,664.8 ± 620.8 (974-2,605)	663.1 ± 430.2 (118-1,677)
Surgery					
	Single adenoma	22	7	6	9
	Multiglandular disease	4	0	1	3
	Carcinoma	3	0	2	1
	Thyroidectomy	18	4	8	6
	Papillary thyroid carcinoma	7	2	4	1
	Single gland volume (mL)	8.8 ± 1.1 (0.2-50.3)	13.1 ± 17.8 (0.2-50.3)	7.6 ± 4.4 (0.6-1.2)	6.2 ± 7.7 (0.9-25.1)
	Intraoperative PTH decay	83.7 ± 8.3 (63.1-93.7)	86.9 ± 2.7 (82.9-89.8)	82.6 ± 9.8 (65.9-93.7)	83.1 ± 9.3% (63.1-93.7)

Note. Data are presented in mean ± SD (min to max); total calcium reference ranges 8.6-10.2 mg/dL; ionized calcium reference ranges 1.24-1.41 mmol/L; PTH reference ranges 15-65 pg/mL; formula for gland volume calculation = L × W × H × 0.52 (mL).

Group 1 had seven patients (1.8%) highly symptomatic for HIHC. They were all admitted through the emergency care unit because of poor health condition. Polyuria and polydipsia occurred in six patients, three had acute renal injury, and two of them needed hemodialysis. Confusion or lethargy occurred in four patients, whereas nausea and vomiting affected three patients. Their mean calcium level (17.4 mg/dL) was statistically higher than the rest of the cohort (15.1 mg/dL, *p* = 0.03).

All had their diagnosis of PHPT established only after admission, within the first 24 hours. They were managed with hydration, diuretics, and pamindronate. Only two of them had a good response to medication with a calcium nadir below 14 mg/dL, and for this reason, their surgery could be delayed until full clinical recovery. One had a moderate response, undergoing surgery after 17 days of admission with serum calcium of 14.3 mg/dL. The remaining four patients had no clinical or laboratorial response and underwent immediate parathyroidectomy (mean of 5 days after hospitalization).

Of these last four unresponsive patients, three patients had renal impairment, and two patients who needed hemodialysis evolved with a progressive clinical deterioration despite an initial clinical improvement. Both patients underwent surgery in extreme adverse condition – one patient inside the intensive care unit because of severe hypotension and extracorporeal membrane oxygenation and the other patient who died after surgery due to cardiac arrest and resuscitation.

Group 2 had nine patients with severe PHPT in which four patients had their PHPT diagnosis established only after admission. Pathological fractures and/or brown tumors hastened hospitalization in six patients. Two patients had underlying clinical conditions that precipitated hospital admission. The last patient was admitted in face of a calcium level of 19.3 mg/dL, despite having kidney stones as the only symptom. One patient who had a spinal brown tumor with neurological symptoms died 30 days after surgery due to pulmonary sepsis.

Except for the patient who died after surgery due to cardiac arrest, all patients in groups 1 and 2 showed fast normalization of calcium levels, followed by unequivocal and rapid clinical improvement.

The remaining 13 patients of group 3 were all successfully treated according to our institutional routine with a scheduled elective surgery.

Considering the 29 patients with calcium level above 14 mg/dL, patients in groups 1 and 2 (55%) required early admission and immediate resolution of their PHPT. Except for one patient who was asymptomatic, 28 patients had at least one classical symptom or sign of acute calcium intoxication. With two deaths related to HIHC, 27 patients had a mean follow-up of 6.6 years. Two patients evolved with end-stage renal disease and secondary hyperparathyroidism. There was no PHPT recurrence.

## DISCUSSION

Hyperparathyroid-induced hypercalcemic crisis is a life-threatening condition that requires immediate medical intervention. Parathyroidectomy is an effective treatment for correcting hypercalcemia even in critically ill patients and should always be considered as an early treatment option.

The incidence in the literature ranges from 2% to 21% ( 4 – 10 ). Despite being the mostly used criterion, relying wholeheartedly on calcium level and symptoms of toxicity for the establishment of HIHC is imprecise and produces variations among publications.

Different calcium levels were adopted in the literature. The criteria varied from 12 mg/dL ( 10 ), 13.5 mg/dL ( 8 ), 14 mg/dL ( 4 , 5 , 7 , 11 ), and 15 mg/dL ( 7 , 12 ). Toxicity is also highly variable and can be represented by a whole myriad of signs and symptoms. Initially, they can mimic regular symptoms of PHPT, such as constipation, dyspepsia, muscle weakness, poor concentration, irritability, and renal stones. They assume more features of HIHC, such as pancreatitis, acute renal injury, lethargy, coma, arrhythmia, and cardiac arrest, only with progression ( 13 ).

Thus, to avoid misinclusion of patients with high serum calcium but no HIHC, we advocated stricter rules for establishing the diagnosis. The inclusion of hospitalization due to poor clinical status as an additional criterion enhances the selection of patients, which will ultimately benefit most from the correct diagnosis. Assuming this condition, our cohort had a 1.8% incidence of HIHC among our entire historical cohort.

More advanced presentation (groups 1 and 2) had a 62% prevalence of female patients in accordance with previous reports ( 4 , 5 , 7 , 9 , 12 ).

The initial clinical support has no literature consensus, but all are aimed at lowering calcium levels, correcting dehydration, increasing renal calcium excretion, and/or decreasing osteoclast activity ( 4 , 5 , 12 ). Protocols can include hydration, diuretics, hemodialysis, bisphosphonate, calcitonin, and calcimimetic among their strategies ( 4 , 12 ). On the basis of medical management, large series achieved the best results with a combination of hydration, diuretics, and bisphosphonate ( 4 , 7 , 9 ), as it was applied in our cohort.

In contrast with Ahmad el al. who stated that HIHC evolves from pre-existing modest PHPT ( 4 ), all our patients with HIHC (group 1) had their diagnosis of PHPT established only after hospitalization. At hospital admission, any patient with symptoms related to HIHC had their calcium levels measured. The observation of hypercalcemia should promptly raise the possibility of PHPT, which should be confirmed with PTH measurements.

After PHPT is established and initial supportive measurements have taken place, localization exams can be performed. In our institution, it is more feasible to perform ultrasound or computed tomography in critically ill patients. Sestamibi is only possible for outpatients. Initial imaging modality should follow the surgeon's best practice and expertise ( 4 ). As diagnosis and localization exams can take several hours, it is reasonable to prioritize medical management while they are being performed.

Parathyroidectomy is the only definitive treatment, and medical management should be considered a preparation for surgery ( 5 , 8 ). Except for one patient who died after surgery, all patients in the cohort showed normal calcium levels and clinical improvement after surgery. Obviously, suitability and comorbidity play a role in the decision making with regard to the timing of surgery.

Due to its uncommon and variable presentation, there is no definition about the ideal moment for performing surgery in HIHC scenario. The first reports advocate surgery only in light of clinical stabilization in order to minimize perioperative complications ( 4 , 9 ). But more recent studies have reported similar success rate even among unstable and unresponsive patients ( 11 , 14 ). Nowadays, most authors support an early surgery as a key to successful treatment that should be performed right after medical optimization in order to prevent further instability and progression ( 4 – 9 , 11 , 12 , 14 ).

In our patients with HIHC (group 1), only two out of seven patients (28.6%) had a good response to medical treatment. They both reached calcium levels below 14 mg/dL and had their surgeries delayed until full clinical recovery. These findings are in accordance with authors who reported that a huge decay in the calcium levels was related to better outcome ( 3 , 5 ).

Regardless of initial clinical treatment option, patients with HIHC require improvement within hours. Progression can lead to oliguria/anuria, coma, and cardiac arrest that can all be lethal ( 5 , 12 ). In our group of HIHC, five out of seven patients (71.4%) had moderate or poor response to initial medical management. Four patients were unresponsive, and two patients had progression that led to one cardiac arrest (and death) and one cardiogenic shock. Besides that, in our public hospital, it is not always possible to have quick access to surgical center and/or specialized surgeons. Clinicians should keep this in mind and avoid the situation of surgery as a last resource, as occurred with these last two patients.

Ziegler and Yu and cols. advocate that surgery should ideally be performed between 24 and 48 hours after hospitalization, particularly in severe cases ( 3 , 12 ). These authors stated that medical response seems to have a clear trend after this period and that poor responders had a sustained high calcium level and were resistant to additional treatments ( 3 , 12 ). Comprehensive analysis of our data agrees with these authors, and patients classified as poor responders appear to have a narrow window of clinical improvement within the first 48 hours, followed by progression and clinical deterioration. Surgery should be planned from patients’ admission and performed right after this initial stabilization.

To our knowledge, only one report cited the worst outcome related to patients requiring hemodialysis ( 12 ) as observed in our study. Anuric patients have an impaired response to hydration and diuretics, mainstays of initial hypercalcemic treatment. The small number of patients in both studies does not allow a definitive conclusion, but patients who require hemodialysis during initial therapy seem to have a higher risk for a poor outcome.

During surgery, 18 (62%) patients had an associated thyroidectomy, but in some patients, it was overlooked due to poor clinical conditions. Laboratorial identification of high values of calcium and PTH should always lead to a suspected diagnosis of parathyroid carcinoma, and for this reason, in bloc resection should be considered ( 15 ). In our cohort, there were 22 (75.9%) patients who had single adenomas; four (13.8%) patients had multiglandular disease; and three (10.3%) patients had carcinomas. The high incidence of carcinoma is expected due to patients’ selection bias ( 4 , 8 , 9 ).

As observed in our cohort, a large parathyroid lesion is a common finding among patients with HIHC ( 5 , 12 ). Despite the initial severeness, long-term follow-up is usually represented by a long eucalemic period with no relapses ( 8 , 9 ). In over 6 years of mean postoperative period, we had no recurrence of PHPT even among multiglandular disease or patients with carcinoma.

The long period of time covered by the data collection may represent a potential limitation to the study. Over 21 years, PHPT management has passed through the advent of new technology, variable laboratory assays, new pathological classifications, and changes in surgical technique.

In summary, we conclude that HIHC is an urgent clinical condition that requires rapid diagnosis and treatments. Clinical supportive measurements should be aimed in lowering calcium levels as fast as possible. Parathyroidectomy is the only definitive therapy that ensures full and long-stand recovery. Surgery should be planned since patient admission and carried out after initial clinical optimization. Care must be taken to postpone surgery beyond 48 hours, due to the risk of rapid clinical deterioration.
